# Prevalence of falls among adult mothers in Saudi Arabia: a cross-sectional study

**DOI:** 10.1186/s12905-023-02760-y

**Published:** 2023-11-09

**Authors:** Yousef M. Alshehre, Sattam M. Almutairi

**Affiliations:** 1 Department of Physiotherapy, Faculty of Applied Medical Sciences, University of Tabuk, 71491 University of Tabuk, Tabuk, 47713 Saudi Arabia; 2 Physiotherapy Department, College of Medical Rehabilitation Science, Qassim University, Buraydah, Saudi Arabia

**Keywords:** Accidental falls, Mothers, Pregnant women, Woman’s health, Prevalence

## Abstract

**Background:**

The prevalence of falls among mothers (18–49 years old) in Saudi Arabia has been overlooked and understudied. Therefore, the study aimed to identify the 1-year prevalence, rate of falls, and consequent injuries among mothers in Saudi Arabia.

**Methods:**

In this cross-sectional study, a self-administered online questionnaire, including sociodemographic data and questions related to the history of falls and consequent injuries during the past 12 months, was disseminated through social media in Saudi Arabia.

**Results:**

A convenience sample of 986 mothers were voluntarily recruited for this study with a median age of 33 years and an interquartile range of 10 years. The 1-year prevalence of falls among mothers was 14.1 % (*n* = 139), and 52.5 % (*n* = 73/139) of the fallers experienced more than one fall. Among mothers who had experienced a fall, 25.4 % (*n* = 33/139) experienced a fall incident during pregnancy. The reported consequences of falls were pain in 37.4 % (*n* = 52/139), muscle and ligament injuries in 7.2 % (*n* = 10/139), and fractures in 2.2 % (*n* = 3/139) of participants. The study’s findings indicate that asthma and high cholesterol level predicts the risk of falls in mothers.

**Conclusions:**

According to our convenience sampling, 14.1% of mothers had experienced one or more falls in the past 12 months. The increased prevalence of falls among this age group of women supports the idea that falls are not only an issue for the older adult population, but fall prevention strategies for this age range are also needed.

## Introduction

Falls and associated injuries are a growing burden, ranking as the second leading cause of years lived with disability globally [1]. One in four individuals annually suffers from a fall, and less than half seek medical attention [2]. The prevalence of falls varies depending on country, sex, and age group [1–3]. Falls varied widely across countries, ranging from 11 to 37.4% of falls annually [4–8]. In Saudi Arabia, previous literature demonstrated that 31.6 to 57.7% of older individuals experienced at least one fall incident in the past year [7–13]. Numerous biological, social, environmental, and behavioural factors may contribute to falls. For example, sex plays a significant role in predicting falls [14]. There is consistent evidence that females are more prone to falls than males, and this rate increases with advanced age [12, 15]. In particular, females have 1.3 times higher fall rates than males [16]. Women account for 58% of nonfatal fall injuries worldwide [17]. Furthermore, women are more likely to develop bone fractures after falls. Data show that women are two times more susceptible to hospitalisation and admission to emergency departments [17].

A 2019 systematic review and meta-analysis evaluated six studies of adults aged ≥60 years in Gulf Cooperation Council countries and reported a high prevalence of falling (46%), with women at a higher risk of falling (60%) than men (42%) [12]. A study conducted in patients aged ≥60 years in Unaizah City indicated that the prevalence of falls was 34.5% in women and 28.5% in men [9]. Another study in Jeddah City reported that 51.3% of older females had a history of falls compared to 39.4% of older males [11]. Previous studies have reported that hormonal changes experienced by women as they age or after menopause may cause an increased incidence of falls [12, 18]. These changes may be linked to a more rapid decline in bone mass compared to men. Sarcopenia, a condition characterised by the loss of skeletal muscle, has been identified as an additional risk factor associated with falls in females [19]. Women have more fear of falls and significant fall injuries than males; therefore, focusing on women is warranted. Furthermore, preventing and treating injuries caused by falls is an important aspect of women's health [20].

Although the risk of falls increases with age, falls are more prevalent among middle-aged individuals, particularly women [21]. Many fall-related risk factors, both modifiable and non-modifiable, that play a role in fall rates have been identified in middle-aged adults [1, 16]. Notably, during postpartum, which can last up to 6 months postdelivery, women undergo emotional and bodily changes, and a few of them may never fully return to the prepregnant state [22, 23]. According to a 2021 Saudi Arabian report released by the General Authority for Statistics, 59.5% of women in Saudi Arabia are between the ages of 18 and 49 years [24]. Owing to the considerable increase in the age-group population, it is imperative to investigate the prevalence of falls among mothers to create effective and efficient prevention and intervention programs. The literature on falls is extensive and largely focuses on older adults; however, less attention has been paid to falls experienced by mothers in the general population, especially those who have delivered a baby. Existing studies have predominantly concentrated on individuals aged 50 years and older, with limited research investigating falls in middle-aged mothers within the age range of 18 to 49 years [21]. Therefore, the present study aimed to estimate the annual frequency and rate of falls, and consequent injuries of falls among mothers aged 18–49 years in Saudi Arabia over the past 12 months. Furthermore, this study aimed to assess the association between history of fall and medical comorbidities.

## Materials and methods

### Study design and participants

This was an online, cross-sectional survey study conducted between January and May 2022. Trained research assistants distributed the survey through social media platforms such as X and WhatsApp among mothers in Saudi Arabia. The participants were selected using a convenience sampling and included women who met the following criteria: 1) aged 18–49 years because the prevalence of fall risk factors tends to increase in women after menopause, typically after the age of 50 [3, 23, 25–27]; 2) able to read and understand the Arabic language independently; and 3) had delivered a baby. This study was reviewed and approved by the Institutional Review Board of the Ministry of Health, Buraydah, Saudi Arabia (Approval No.:1443-225277). The study was conducted in accordance with approved guidelines and all participants were informed about the study's purpose. Participants who agreed to participate signed an informed consent form before completing the survey.

### Questionnaire

The authors developed the questionnaire that underwent expert review. This self-administered online survey consisted of three main parts: personal sociodemographic data, presence comorbidities, and questions concerning the prevalence and the number of falls per person, and the consequences of falls among mothers over the past 12 months.

The sociodemographic characteristics part covered several factors, such as age, height in centimetres (cm), weight in kilograms (kg), and body mass index (BMI), calculated by dividing weight in kg by height in m^2^. It also included information on nationality, employment status, educational level, current city of residence, marital status, smoking status, pregnancy status, yearly income, and medical comorbidities. The medical comorbidities included the following conditions: asthma, diabetes mellitus, high blood pressure, high cholesterol level, heart diseases, arthritis, osteoporosis, cancer and tumors, hypothyroidism, liver disease, psychological disorders, anemia, and colon disorders.

The part on the prevalence of falls captured data on the frequency of falls within the past 12 months and the resulting consequences. The study adapted the World Health Organization's definition of falls, which is ‘an event which results in a person coming to rest inadvertently on the ground, floor, or other lower level.’ [1]

To ensure the questionnaire's validation, it was first tested with a sample of eligible mothers (*n* = 15). This allowed the authors to assess the survey's design, gather feedback on the survey’s language and usability, and make any necessary adjustments. Minor modifications were made to the questions based on the feedback received to ensure a clear understanding and obtain the required information. The questionnaire included a consent section at the beginning.

Regarding falls, participants were asked, ‘Have you experienced any falls within the last 12 months?’ If the response given was affirmative, the participants were further asked, ‘How many times have you fallen in the past 12 months?’ For those who reported one or more falls, additional questions were posed regarding the resulting injuries. The consequences of falls were categorized into four groups: fracture, muscle and ligament injury, pain, and no injury. Participants were categorized into (faller) has at least one fall, and (non-faller) has no history of fall. These questions aimed to estimate the prevalence of women who reported falls over the past 12 months, evaluate the rate of falls for each participant who experienced a fall, and assess the resulting injuries from those falls [15].

### Statistical analysis

The main outcomes of this study focused on the history of falls and the number of falls experienced by participants. The data collected from the questionnaires were analysed statistically using the IBM Statistical Package for Social Science software (version 28, SPSS Inc., Chicago, Illinois, USA). Demographic characteristics and medical comorbidities were reported as mean (m) or median, standard deviation (SD) or interquartile range (IQR), count (n), frequency (f), and percentage (%). To compare fallers and non-fallers, statistical tests such as chi-square or Fisher's exact tests were used for categorical variables, while the independent t-test was used to analyse continuous variables.

To determine the association between medical comorbidities and fall history (differentiating between fallers and non-fallers), a binary logistic regression analysis was conducted. The enter method was used to include all variables simultaneously in the model. The selection of variables for inclusion was based on their clinical relevance to falls and their significance level in the unadjusted model. Medical comorbidities were entered as predictors, while fall status (yes for fallers and no for non-fallers) was the dependent variable (outcome). Odds ratios (OR) with 95% confidence intervals (95% CIs) were calculated for each medical comorbidity. In the primary analysis, potential confounders were controlled for, including age, education, and BMI.

Few answers on income range, height, and residential region entered were not applicable (N/A) because few participants preferred not to declare or incomplete data were provided. Therefore, missing variables were handled via case-wise deletion.

## Results

### Demographics and descriptive

The study included a total of 986 participants, all of whom were mothers. Their ages ranged from 18 to 49 years, with a median of 33 years and an interquartile range of 10 years. Most participants (95.6%) were of Saudi nationality. The average BMI was 28.6 ± 12.6 kg/m^2^. Among the participants, 45.5% (*n* = 449) were homemakers, and the majority, 96.1% (*n* = 948) were married. More than half of the participants (59%; *n* = 582) had a bachelor's degree. Only 0.5% of the participants were smokers. Additional sociodemographic characteristics of fallers and non-fallers are presented in Table 1. The most common comorbidities among the participants were asthma (6.3%; *n* = 62), diabetes mellitus (4.3%; *n* = 42), high blood pressure (4.1%; *n* = 40), and high cholesterol levels (3.5%; *n* = 35). A complete list of medical comorbidities can be found in Table 2.

**Table 1:** Participants’ sociodemographic characteristics (*n* = 986)

Demographic characteristics	Total sample n (%)	Faller n (%)	Non-faller n (%)	*P*-value
Nationality				0.188
Saudi	943 (95.6%)	130 (93.5%)	813 (96%)	
Non-Saudi	43 (4.4%)	9 (6.5%)	34 (4%)	
Age group (years)				0.722
18-25	78 (7.9%)	12 (8.6%)	66 (7.8%)	
26-30	223 (22.6%)	34 (24.5%)	189 (22.3%)	
31-35	224 (22.7%)	24 (17.3%)	200 (23.6%)	
36-40	193 (19.6%)	30 (21.6%)	163 (19.2%)	
41-45	142 (14.4%)	21 (15.1%)	121 (14.3%)	
46-49	126 (12.8%)	18 (12.9%)	108 (12.8%)	
BMI, Kg/m^2^				0.709
m ± SD (missing = 26)	28.6 ± 12.6	29 ± 14.8	28.6 ± 12.2	
Marital status				0.518
Married	948 (96.1%)	136 (97.8%)	812 (95.9%)	
Divorced	22 (2.2%)	2 (1.4%)	20 (2.4%)	
Widowed	16 (1.6%)	1 (0.7%)	15 (1.8%)	
Educational level				0.437
Higher education	753 (76.3%)	2 (1.4%)	8 (0.9%)	
Master	29 (2.9%)	7 (5%)	22 (2.6%)	
Bachelor	582 (59%)	86 (61.9%)	496 (58.6%)	
Diploma	132 (13.4%)	14 (10.1%)	118 (13.9%)	
Primary education	227 (23%)	22 (15.8%)	143 (16.9%)	
No formal education	6 (0.6%)	1 (0.7%)	5 (0.6%)	
Annual income (SR)				0.647
< 48000	325 (33%)	41 (29.5%)	284 (33.5%)	
48000 -71999	222 (22.5%)	37 (26.6%)	185 (21.8%)	
72000 - 95999	104 (10.5%)	17 (12.2%)	87 (10.3%)	
96000 - 1119999	90 (9.1%)	12 (8.6%)	78 (9.2%)	
> 120000	152 (15.4%)	22 (15.8%)	130 (15.3%)	
Missing	93 (9.4%)	10 (7.2%)	83 (9.8%)	
Employment status				0.636
Homemaker	449 (45.5%)	64 (46%)	385 (45.5%)	
Full time	237 (24%)	34 (24.5%)	203 (24%)	
Not working and looking for a job	167 (16.9%)	21 (15.1%)	146 (17.2%)	
Retired	50 (5.1%)	5 (3.6%)	45 (5.3%)	
Student	42 (4.3%)	8 (5.8%)	34 (4%)	
Part time	41 (4.2%)	7 (5%)	34 (4%)	
Smoking				0.095
Yes	5 (0.5%)	2 (1.4%)	3 (0.4%)	
No	981 (99.5%)	137 (98.6%)	844 (99.6%)	

*n* count; *%* percentage, *BMI* Body mass index, *SR* Saudi Riyal, *m* mean, *SD* standard deviation, *P* > 0.05 not significant

**Table 2:** Medical comorbidities among mothers in Saudi Arabia (*n* = 986)

Chronic conditions	Overall sample n (%)	Faller n (%)	Non-faller n (%)	*P*-value
Asthma	62 (6.3%)	14 (10.1%)	48 (5.7%)	0.047^*^
Diabetes mellitus	42 (4.3%)	9 (6.5%)	33 (3.9%)	0.163
High blood pressure	40 (4.1%)	7 (5%)	33 (3.9%)	0.528
High cholesterol levels	35 (3.5%)	9 (6.5%)	26 (3.1%)	0.044^*^
Heart disease	9 (0.9%)	2 (1.4%)	7 (0.8%)	0.482
Arthritis	13 (1.3%)	0	13 (1.5%)	0.141
Osteoporosis	12 (1.2%)	3 (2.2%)	9 (1.1%)	0.275
Cancer and tumors	7 (0.7%)	1 (0.7%)	6 (0.7%)	0.989
Hypothyroidism	24 (2.4%)	2 (1.4%)	22 (2.6%)	0.411
Liver disease	1 (0.1%)	0	1 (0.1%)	0.685
Psychological disorders	2 (0.2%)	1 (0.7%)	1 (0.1%)	0.144
Anemia	8 (0.8%)	1 (0.7%)	7 (0.8%)	0.896
Colon disorders	3 (0.3%)	0	3 (0.4%)	0.482

*n* count; *%* percentage, *P* > 0.05 not significant; Chi-square test *-* ^*^ *P* < 0.05 significant

The study found that 14.1% (*n* = 139) of the participants had experienced at least one fall in the past 12 months and were classified as fallers, while 85.9% (*n* = 847) had not experienced any falls and were classified as non-fallers. The reported number of falls during the 12 months ranged from 1 to 10. Among the fallers, more than half (52.5%; *n* = 73/139) had experienced multiple falls. Specifically, 38.2% (*n* = 53/139) had fallen two to three times, 11.5% (*n* = 16/139) had fallen four to five times, and only 2.8% (*n* = 4/139) had fallen more than five times. The consequences of the falls included pain (37.4%; *n* = 52/139), muscle and ligament injuries (7.2%; *n* = 10/139), and fractures (2.2%; *n* = 3/139). Additionally, 33 participants (25.4%) reported falling during their pregnancy.

Chi-square tests were conducted to assess potential risk factors for falls, and the presence of bronchial asthma and high blood cholesterol were found to be marginally correlated with the history of falls among the included Saudi women (p = 0.047 and 0.044, respectively). The results of the binary logistic regression analysis, which examined the association between medical comorbidities and falls (faller versus non-faller), are presented in Table 3. The OR and associated 95% CIs are also reported in Table 3. Fallers were found to be more likely to have asthma (OR: 1.88; 95% CI: 1.0–3.52; p = 0.04) and high cholesterol levels (OR: 2.39; 95% CI: 1.10–5.41; p = 0.035).

**Table 3:** The association between history of falls and medical comorbidities

Medical comorbidities	OR	95% CI	*P*-value
Asthma	1.88	1.003–3.53	0.049^*^
Diabetes mellitus	1.96	0.892–4.325	0.094
High blood pressure	1.37	0.577–3.289	0.471
High cholesterol levels	2.39	1.063–5.410	0.035^*^
Heart diseases	2.04	0.412–10.13	0.382
Osteoporosis	2.14	0.566–8.096	0.262
Cancer and tumors	1.06	0.126–9.020	0.95
Hypothyroidism	0.54	0.127–2.37	0.42
Psychological disorders	6.03	0.374–97.56	0.205
Anemia	0.921	0.112–7.583	0.93

*OR* Odds ratio, Covariates included age, education, Body mass index, and medical comorbidities. *CI* confidence intervals, Binary logistic regression test - ^*^ *P* < 0.05 significant

## Discussion

This study investigated the prevalence of falls and associated medical comorbidities among mothers in Saudi Arabia. The findings of the present study showed that 14.1% of mothers in Saudi Arabia had experienced at least one fall in the past 12 months, with 52.5% having experienced more than one fall. The prevalence of falls in this study was lower than that reported in other studies in Saudi Arabia that evaluated older Saudi citizens in Riyadh (49.9%) [12], older patients from primary healthcare centres in Unaizah City in Qassim Province (31.6%) [9], as well as community-dwelling older adults in Jeddah (47.4%) [11]. Globally, the Australian longitudinal study on women's health analysed data from mid-age women (50–64 years old) and investigated falls and related health factors, and found a significant prevalence of falls, with approximately 20.5% to 30.7% of women reporting falls in the previous 12 months across different survey intervals [21]. Notably, the age of the participants in the present study was lower than that in other studies, which could partly explain the lower prevalence of falls observed. However, this result confirms that falls are common among women aged 18–49 years. A possible explanation for this finding is that younger women are more likely to be physically active and engaged, predisposing them to fall [26].

The present study revealed a high prevalence of falls among women during pregnancy with approximately a quarter (25.4%) of women reporting falling at least once during pregnancy. These results agreed with those of international studies from Nigeria (32.5%) [27], Turkey (31.9%) [28], and the USA (27%) [29]. The rate of maternal falls during pregnancy is comparable to that of a 65-years-old women [30]. The literature highlights pregnancy-related hormonal, physiological, anatomical, and postural changes associated with falls during pregnancy [22, 31]. These changes during pregnancy challenge postural equilibrium and increase the risk of falls. Consistent with our findings, pregnant women aged <30 years have been found to have a higher risk of falling [31].

Among the mothers who fell, 52.5% reported recurrent falls in the past year. Recurrent fallers may develop fear of falling which is associated with activity restriction and reduced quality of life [32]. Several studies indicated that fear of falls is higher among women [20, 32]. The fear of falling is a serious health issue independent of falls. A previous study reported that 20% of participants without a recent or recurrent fall developed a fear of falling [20]. The fear of falling can create physical, psychological, and functional limitations. Notably, several recommended therapies, including physical therapy, can help reduce the fear of falling [33].

In this study, women who reported asthma or high cholesterol levels had a 1.88 and 2.39 OR higher risk of falls than those who did not. A possible explanation is that participants with asthma may have difficulty with physical activity and function which in turn are related to an increased risk of falls [34, 35]. Furthermore, asthma is related to obesity, which is associated with an increased risk of falls [36, 37]. This study identified novel predictors of falls among mothers. Several studies have found that the incidence of asthma decreases after menopause and is not considered a risk factor for falls in older women [38, 39]. These results emphasize that falls are influenced by a multifaceted and evolving combination of risk factors. The findings corroborate and expand upon existing research, which has consistently shown that various indicators of poor health are associated with a higher likelihood of falling among mid-aged women. Numerous factors have been identified as risks for falls in mid-age women. For example, a higher fall risk was observed in overweight and obese women compared to those with a normal weight. Impaired vision and diminished physical functioning consistently heightened the chances of falling. Furthermore, factors like depression, urinary incontinence, joint problems, severe fatigue, osteoporosis, and hormone replacement therapy were also linked to falls in this particular group [21].

Numerous clinical practice guidelines have been developed for fall prevention and management in different settings including communities, nursing homes, and acute care [33]. Studies have reported that focusing fall prevention interventions on adults aged ≥65 years reduces the fall rate by approximately 30% [3]. A recent systematic review summarised the findings of 15 high-quality practice guidelines for fall prevention and management among adults aged ≥60 years and indicated that most clinical practice guidelines for fall prevention and management strongly recommend physical exercise interventions and physical therapy referrals as key elements in fall prevention [8]. Exercise interventions have been found to reduce the rate of falls in older adults by approximately 25%. Exercise intervention programs are cost-effective fall prevention approaches, primarily involving functional, balance, and resistance exercises. However, the target population for these guidelines was mainly adults aged ≥60 years. Clinical practice guidelines for mothers lack recommendations for fall prevention. It is imperative to establish fall prevention clinical practice guidelines considering sex and age groups. Attention is needed to prevent falls in women, especially after delivery and during e postpartum.

## Limitations

This study had some limitations. First, the occurrence of falls relied on the participants’ memory, which was prone to recall bias. Second, factors such as standardize questionnaire, functional status, fear of falling, balance, lower extremity strength, and medication use were not measured, which may have affected the prevalence estimates. Third, it is possible that falls were underreported, particularly in female populations which may lead to an underestimation of the true incidence of falls. Non-probability sampling methods utilizing social media have their advantages in terms of convenience and cost-effectiveness. However, they are often associated with limitations in terms of generalisability and potential biases. Therefore, the generalisability of our findings is limited. Lastly, the sample size calculation was not conducted in this study. Future studies should focus on promotion of prevention strategies and education for women to reduce the incidence of falls in this population.

## Conclusion

In contrast to older adults, little is known about the prevalence of falls among mothers aged 18–49 years. In this study, 14.1% of the mothers had experienced one or more falls in the past 12 months in Saudi Arabia. To the best of our knowledge, no previous studies have assessed the prevalence of falls among women who delivered babies, making it difficult to compare data. The prevalence values obtained in the present study will help establish appropriate strategies to reduce falls and fall-related injuries in this age group.

## Data Availability

The datasets analysed during the present study are available from the corresponding author upon reasonable request.

## Abbreviations

SD: Standard deviation
cm: centimetres
kg: kilograms
BMI: Body mass index
m: mean
IQR: Interquartile range
N: Count
f: frequency
%: Percentage
OR: Odds ratios
CIs: Confidence intervals
